# Early Diagnosis of Nonconvulsive Status Epilepticus Recurrence with Raw EEG of a Bispectral Index Monitor

**DOI:** 10.1155/2018/1208401

**Published:** 2018-09-12

**Authors:** Aristide Ntahe

**Affiliations:** Département d'Anesthésie-Réanimation, Hôpital Saint-Louis, Assistance Publique-Hôpitaux de Paris, 1 Avenue Claude Vellefaux, 75010 Paris, France

## Abstract

**Background:**

Seizures are frequent in ICU and their diagnosis is challenging, often delayed or missed. Their diagnosis requires a conventional EEG recording. When cEEG is not available, there is no consensus on how patients should be monitored when there is high risk of seizure. This case illustrates how a bispectral index monitor allowed an early diagnosis of an NCSE recurrence.

**Case Presentation:**

A NCSE was diagnosed at the admission. cEEG was not available and then a bispectral index (BIS) monitor was placed and processed parameters were monitored as usual. During the first and second day, both conventional and BIS's EEG showed patterns of burst suppression and the BIS value varied between 25 and 35 while the suppression ratio (SR) varied between 20 and 35. On the third day, while hypnotic drugs were withdrawn progressively, raw EEG of the BIS monitor showed spikes, spikes waves, and polyspikes without significant variation of BIS and SR values. Even if processed parameters stayed between their usual ranges, the typical aspect of the real time EEG raised concern for NCSE recurrence. An unplanned conventional EEG recording was urgently requested, and the diagnosis was confirmed and treated.

**Conclusion:**

Primitive and secondary brain injuries can lead to seizures which are often purely electrical. Even though BIS monitors cannot substitute the conventional EEG, processed parameters and raw EEG should be always analysed jointly. In the present case, seizure was suspected only on the aspect of real time EEG which showed spikes, spikes waves, and polyspikes.

## 1. Background

Hypoxic-ischaemic brain injury is one of the most feared complications following cardiac arrest. If present, the incidence of seizures and status epilepticus is high and associated with poor outcome [1]. Managing status epilepticus without continuous EEG (cEEG) is very challenging especially if it is a nonconvulsive status epilepticus (NCSE). Bispectral index (BIS) is one among other technologies used to monitor hypnotic drug's effect in operating room and in intensive care units (ICU). BIS monitors display 2 numbers named BIS and suppression ratio (SR) which are derived from analysis of frontal EEG signal by a single sensor (with multiples electrodes) placed on the forehead. This case showed how the raw EEG signal of BIS monitor allowed a rapid diagnosis of a NCSE recurrence.

## 2. Case Presentation

A 75-year-old woman was admitted in ICU after a cardiac arrest with return of spontaneous circulation, caused by tension pneumothorax. Now-flow and low-flow were 1 minute and 17 minutes, respectively. She was admitted 1 hour after the event, without sedation. Lungs were mechanically ventilated, the pleural drain was in place, pulse oximetry was 100% /FiO2 0.5, arterial pressure and heart rate were 160/70 mmHg and 110bpm, respectively, with norepinephrine at an infusion rate of 1mg/h, and temperature and glycaemia were 36.5°C and 8 mmol/L, respectively. There was no any sign of pneumothorax. Fever was actively treated without inducing hypothermia.

She was unconscious (Glasgow Coma Scale: 3/15), with a conserved bilateral photomotor reflexes. She had intermittent bilateral ocular revulsion and bilateral shoulders tremor. Propofol was initiated by a bolus followed by a continuous infusion and the movements of the eyes and shoulders ceased immediately. One hour later a 13 channels EEG (Figure S1 in supplementary figures) diagnosed a NCSE as a pattern of generalized periodic spike-waves evolving in generalized rhythmic spike-waves at 1 Hz with high amplitude (> 200 *µ*V), without response to stimulation. Midazolam was initiated by a bolus followed by a continuous infusion which permitted to achieve a burst suppression pattern (Figure S2 in supplementary figures). A 4-channel sensor connected to BIS VISTA^®^ monitor was placed, in order to monitor the two processed parameters and showed an isoelectric signal (Figure S3 in supplementary figures).

On day 2 Clobazam and levetiracetam were added to ensure a bridging between IV and oral antiepileptic drugs. Conventional EEG recording showed a pattern of burst suppression and raw EEG from BIS monitor showed an isoelectric signal.

**Figure 1:**
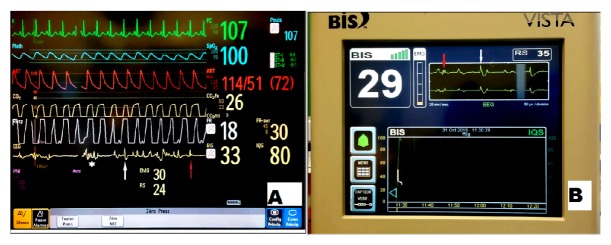
PANEL A: 2 channels sensors (BIS Quatro) connected to a Philips module: real time EEG shows polyspikes (white star), spikes (white arrow), and spike-wave (red arrow). IQS = signal quality index. RS= suppression ratio. PANEL B: 2-channel sensor (BIS Quatro) connected to a BIS VISTA module: real time EEG shows spike-wave (red arrow) and spike (white arrow). IQS = signal quality index. RS= suppression ratio.

On day 3, the 4-channel sensor was replaced by a 2-channel sensor which was connected to patient's bedside monitoring. Propofol and midazolam were both decreased progressively. Few hours later (Figure 1, video 1, and figure S4), while there were no any abnormal movements, raw EEG of BIS monitor connected to a Philips BIS module [panel A] and secondarily to a BIS VISTA module [panel B] showed a pattern of high voltage with irregular morphology, alternating with isoelectric signal, evoking spikes (white arrows), spike-waves (red arrows), and polyspikes (white star). BIS and SR values did not show significant variations.

Although it was not planned at that moment to request a conventional EEG recording, given the high suspicion of NCSE recurrence, the neurophysiology team was urgently contacted and the NCSE recurrence was confirmed and treated. The conventional EEG showed continuous generalized rhythmic spikes and spike-waves, sharply countered, of medium amplitude at 1-1.5 Hz (Figure S5 in supplementary figures). Unfortunately the patient died on the fifth day.

## 3. Discussion

The context of sudden brain aggression, the bilateral stereotyped movements, the EEG pattern, the clinical and EEG responses following propofol, and midazolam injections and withdrawal are all in favour of a NCSE, according to recent definitions [2].

Seizures are frequent in ICU, independently of the presence of primitive brain lesion, and are subclinical in the majority of cases [3–5]; this put the ICU's patients at high risk for secondary brain injury, poor neurologic outcome, and increase mortality [6–8]. Diagnosis of nonconvulsive seizures or status epilepticus can be very challenging and is often delayed or simply missed. One reason is that there are multiple causes of altered mental status in ICU; the second major reason is availability of EEG equipment and interpreting staff. In 2015 the American clinical neurophysiology society issued recommendations on the use of critical care cEEG, focusing on the importance of each care centre to have a program development and improvement and indicating the contexts in which patients should be monitored with a cEEG [9]. According to those recommendations, patients suffering from hypoxic-ischaemic brain injury should be monitored with cEEG.

When cEEG is not available, there is no consensus on how patients should be monitored when there is high risk of seizure. Simplified bedside EEG monitors are more available and offer a real time analysis of brain function. Most of them display one or more channels continuous EEG and their diagnostics performance is directly correlated with the numbers of electrodes used. Average sensitivity is 68% with 4 channels montage [10] and can reach 92.5% with seven electrodes montage [11]. Users should keep in mind that all brain areas are not covered and that brief seizures can be easily missed.

Other bedside monitors can provide quantitative EEG(qEEG) which facilitates interpretation of prolonged EEG recording. Among them, those which provide amplitude-integrated EEG (aEEG) are widely used in clinical practice especially in pediatric ICUs but studies are scarce in adult ICUs. In one study, using a 1-channel montage, authors found a sensitivity of 40% for the identification of seizure by nonexperts ICUs physicians [12]. Another type of quantitative EEG frequently used is compressed spectral array, which can identify seizure patterns with a very good accuracy [13, 14]. However qEEG do not allow instantaneous diagnosis of seizure.

In the case presented here, NCSE was diagnosed at the admission with a conventional EEG. Because a cEEG is not available in our centre, a BIS monitor was placed. BIS monitoring is based on EEG analysis and monitors usually display at the same time a simplified frontal EEG signal and a BIS value between 0 and 100. The BIS value is derived from correlation of the phases between frequency components of the EEG. These monitors also display a SR value which corresponds of the percentage of time in which the EEG is isoelectric over a 63 seconds period.

In ICU, BIS monitors are used in different contexts when patients have intracranial hypertension BIS and SR values are used to titrate barbiturate treatment [10, 11]; when patients have refractory status epilepticus, BIS and SR values are used to guide the depth of sedation if cEEG is not available because there is a strong correlation between BIS and SR values and the burst rate monitored with conventional EEG [12, 13].

The reliability of BIS and SR values depends entirely on a good EEG signal quality, but in routine clinical practice, physicians tend to focus essentially on this two processed parameters. In the present case neither BIS nor SR values changed markedly at the moment the real time EEG started to show seizure patterns, which means that the NCSE recurrence could have been missed or diagnosed with delay. The diagnostic value of the real time EEG of BIS monitor is high because it diagnoses well a recruiting rhythm, spikes, and spikes waves during generalized tonic-clonic seizures [15].

Even though BIS monitors are easy to handle, learning how to interpret EEG signal is a lengthy process, but the benefits for patients are important, because the neurological outcome depends on rapid diagnosis and treatment.

It is important to remember that BIS monitors cannot substitute the conventional EEG, but when a BIS monitor is used, processed parameters and raw EEG should be analysed jointly, and when a rhythm and/or amplitude variations appear on the real time EEG, seizure should be sought.

## 4. Conclusion

In ICU, primitive and secondary brain injuries can lead to seizures which are often purely electrical. When a bispectral index monitor is used, real time EEG should be monitored and interpreted according to the context to detect signs of seizure, even if processed parameters values are unremarkable.

## Data Availability

The data (full images and video, 30 min EEG recordings, and pdf format from BISvista module) are available from the corresponding author upon request.

## List of Abbreviations

aEEG: Amplitude-integrated EEG
BIS: Bispectral index
cEEG: Continuous electroencephalogram
EEG: Electroencephalogram
ICU: Intensive care unit
NCSE: Nonconvulsive status epilepticus
qEEG: Quantitative EEG
SR: Suppression ratio.

## Supplementary Materials

Supplementary Materials I expect the case report to have a high pedagogical value, so there are one principal figure (Figure 1) and one principal video (video 1). There are 5 supplementary figures (figure S1-figure S2-figure S3-figure S4-figure S5): each one is supposed to help the editorial board to confirm the accuracy and the relevance of the case report. Also they will help readers if necessary. (i)** Figure S1** is a conventional electroencephalogram recorded at the admission of the patient which confirmed the nonconvulsive status epilepticus. (ii)** Figure S2** is as the figure S2, a conventional electroencephalogram recorded few hours later, which confirmed that the nonconvulsive status epilepticus was well treated. (iii)** Figure S3** is an electroencephalogram, recorded on day 2, which is displayed by a BISvista monitor connected to a bilateral sensor. (iv)** Figure S4**, as figure S3, is an electroencephalogram, recorded on day 3, which is displayed by a BISvista monitor connected to unilateral sensor. (v)** Figure S5**, as figures S1 and S2, is a conventional electroencephalogram recorded on day 3, which confirmed the nonconvulsive status epilepticus recurrence. The complete files (30 minutes EEG recording for each supplementary figure) are available if they are needed.** Figure S1**: Day 1: 10-20 system EEG: diagnosis of NCSE.** Figure S2**: Day 1: 10-20 system EEG: burst suppression.** Figure S3**: Day 2: PDF format generated by BIS VISTA module: burst suppression 4 channels sensor EEG.** Figure S4**: Day 3: PDF format generated by BIS VISTA module: spikes, spikes waves, and 2-channel sensor.** Figure S5**: Day 3: 10-20 system EEG: diagnosis of NCSE recurrence.
