# Erratum to “Early Diagnosis of Nonconvulsive Status Epilepticus Recurrence with Raw EEG of a Bispectral Index Monitor”

**DOI:** 10.1155/2018/7147626

**Published:** 2018-12-16

**Authors:** Aristide Ntahe

**Affiliations:** Département d'Anesthésie-Réanimation, Hôpital Saint-Louis, Assistance Publique-Hôpitaux de Paris, 1 Avenue Claude Vellefaux, 75010 Paris, France

In the article titled “Early Diagnosis of Nonconvulsive Status Epilepticus Recurrence with Raw EEG of a Bispectral Index Monitor” [1], additional references should have been cited, included in the text below as references 16-18 [2–4]. Accordingly, the sixth and seventh paragraphs in Discussion should read as follows:

In ICU, BIS monitors are used in different contexts: when patients have intracranial hypertension, BIS and SR values are used to titrate barbiturate treatment [16, 17]; when patients have refractory status epilepticus, BIS and SR values are used to guide the depth of sedation if cEEG is not available because there is a strong correlation between BIS and SR values and the burst rate monitored with conventional EEG [18].

The reliability of BIS and SR values depends entirely on a good EEG signal quality, but in routine clinical practice, physicians tend to focus essentially on this two processed parameters. In the present case, neither BIS nor SR values changed markedly at the moment the real time EEG started to show seizure patterns, which means that the NCSE recurrence could have been missed or diagnosed with delay. The diagnostic value of the real time EEG of BIS monitor is high because it well diagnoses a recruiting rhythm, spikes, and spikes waves during generalized tonic-clonic seizures [15].
