# Supplementary figures and images for: Early Diagnosis of Nonconvulsive Status Epilepticus Recurrence with Raw EEG of a Bispectral Index Monitor
Source: Case Rep Crit Care. 2018 Sep 12;2018:1208401. doi: 10.1155/2018/1208401 (PMC6157206; doi:10.1155/2018/1208401)

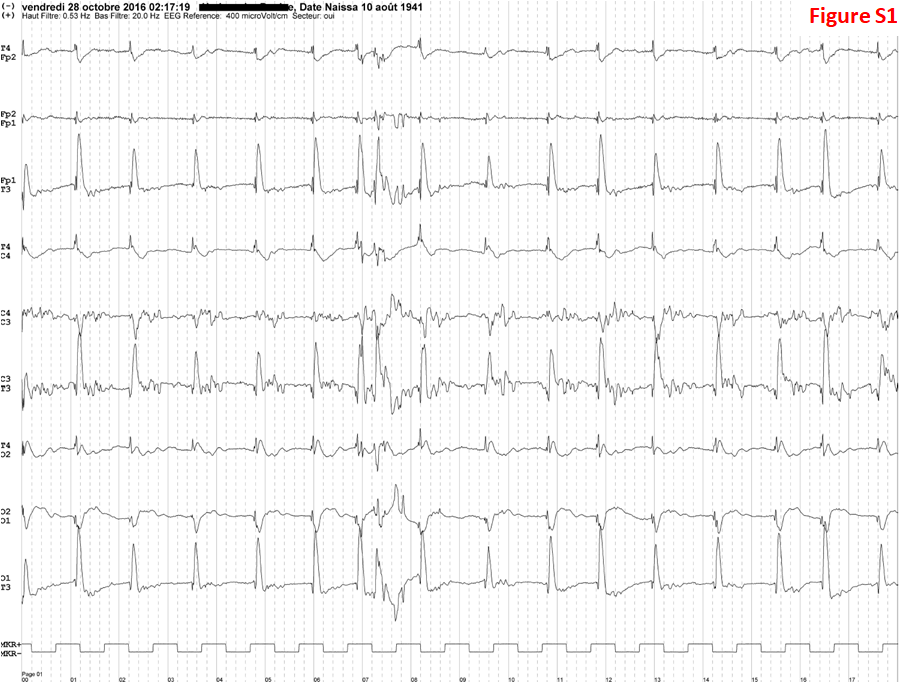

Supplement: Supplementary Materials — I expect the case report to have a high pedagogical value, so there are one principal figure (Figure 1) and one principal video (video 1). There are 5 supplementary figures (figure S1-figure S2-figure S3-figure S4-figure S5): each one is supposed to help the editorial board to confirm the accuracy and the relevance of the case report. Also they will help readers if necessary. (i) Figure S1 is a conventional electroencephalogram recorded at the admission of the patient which confirmed the nonconvulsive status epilepticus. (ii) Figure S2 is as the figure S2, a conventional electroencephalogram recorded few hours later, which confirmed that the nonconvulsive status epilepticus was well treated. (iii) Figure S3 is an electroencephalogram, recorded on day 2, which is displayed by a BISvista monitor connected to a bilateral sensor. (iv) Figure S4, as figure S3, is an electroencephalogram, recorded on day 3, which is displayed by a BISvista monitor connected to unilateral sensor. (v) Figure S5, as figures S1 and S2, is a conventional electroencephalogram recorded on day 3, which confirmed the nonconvulsive status epilepticus recurrence. The complete files (30 minutes EEG recording for each supplementary figure) are available if they are needed. Figure S1: Day 1: 10-20 system EEG: diagnosis of NCSE. Figure S2: Day 1: 10-20 system EEG: burst suppression. Figure S3: Day 2: PDF format generated by BIS VISTA module: burst suppression 4 channels sensor EEG. Figure S4: Day 3: PDF format generated by BIS VISTA module: spikes, spikes waves, and 2-channel sensor. Figure S5: Day 3: 10-20 system EEG: diagnosis of NCSE recurrence. [file 1208401.f1.zip › figure S1_CRICC_2459587.tif]

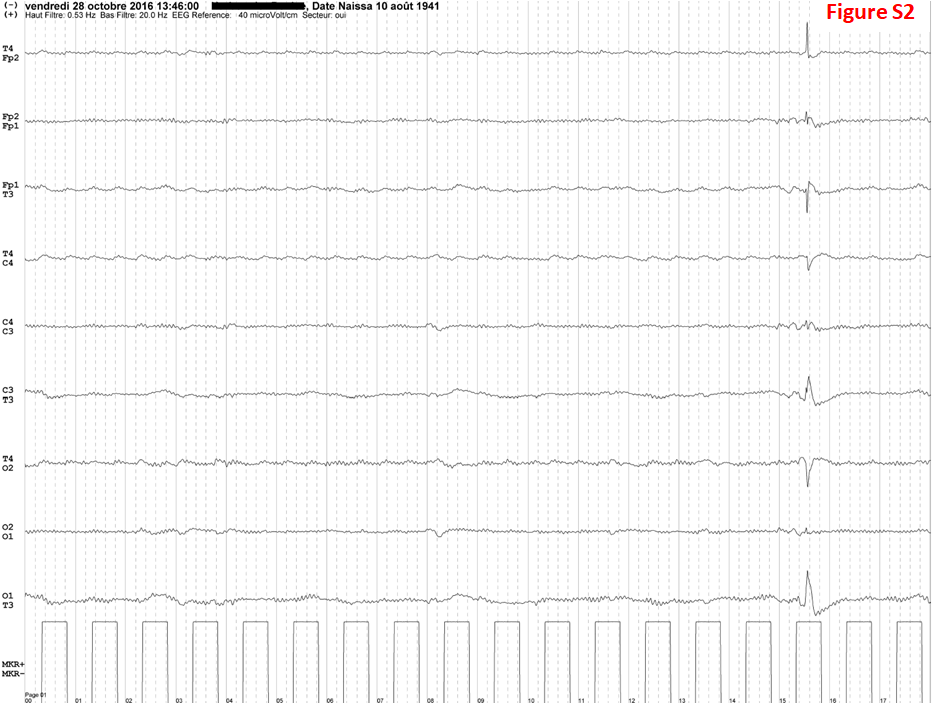

Supplement: Supplementary Materials — I expect the case report to have a high pedagogical value, so there are one principal figure (Figure 1) and one principal video (video 1). There are 5 supplementary figures (figure S1-figure S2-figure S3-figure S4-figure S5): each one is supposed to help the editorial board to confirm the accuracy and the relevance of the case report. Also they will help readers if necessary. (i) Figure S1 is a conventional electroencephalogram recorded at the admission of the patient which confirmed the nonconvulsive status epilepticus. (ii) Figure S2 is as the figure S2, a conventional electroencephalogram recorded few hours later, which confirmed that the nonconvulsive status epilepticus was well treated. (iii) Figure S3 is an electroencephalogram, recorded on day 2, which is displayed by a BISvista monitor connected to a bilateral sensor. (iv) Figure S4, as figure S3, is an electroencephalogram, recorded on day 3, which is displayed by a BISvista monitor connected to unilateral sensor. (v) Figure S5, as figures S1 and S2, is a conventional electroencephalogram recorded on day 3, which confirmed the nonconvulsive status epilepticus recurrence. The complete files (30 minutes EEG recording for each supplementary figure) are available if they are needed. Figure S1: Day 1: 10-20 system EEG: diagnosis of NCSE. Figure S2: Day 1: 10-20 system EEG: burst suppression. Figure S3: Day 2: PDF format generated by BIS VISTA module: burst suppression 4 channels sensor EEG. Figure S4: Day 3: PDF format generated by BIS VISTA module: spikes, spikes waves, and 2-channel sensor. Figure S5: Day 3: 10-20 system EEG: diagnosis of NCSE recurrence. [file 1208401.f1.zip › figure S2_CRICC_2459588.tif]

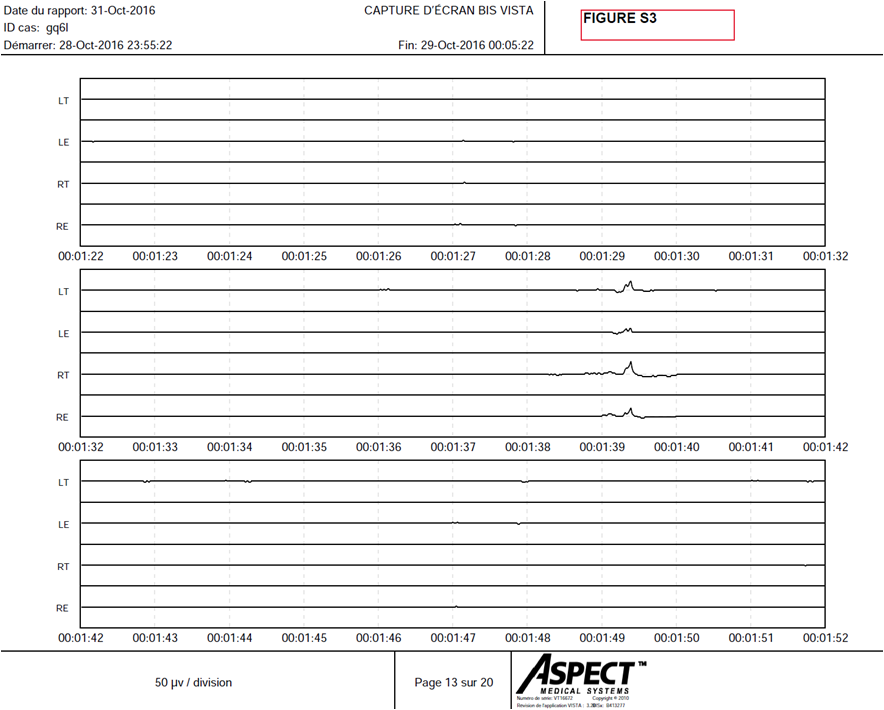

Supplement: Supplementary Materials — I expect the case report to have a high pedagogical value, so there are one principal figure (Figure 1) and one principal video (video 1). There are 5 supplementary figures (figure S1-figure S2-figure S3-figure S4-figure S5): each one is supposed to help the editorial board to confirm the accuracy and the relevance of the case report. Also they will help readers if necessary. (i) Figure S1 is a conventional electroencephalogram recorded at the admission of the patient which confirmed the nonconvulsive status epilepticus. (ii) Figure S2 is as the figure S2, a conventional electroencephalogram recorded few hours later, which confirmed that the nonconvulsive status epilepticus was well treated. (iii) Figure S3 is an electroencephalogram, recorded on day 2, which is displayed by a BISvista monitor connected to a bilateral sensor. (iv) Figure S4, as figure S3, is an electroencephalogram, recorded on day 3, which is displayed by a BISvista monitor connected to unilateral sensor. (v) Figure S5, as figures S1 and S2, is a conventional electroencephalogram recorded on day 3, which confirmed the nonconvulsive status epilepticus recurrence. The complete files (30 minutes EEG recording for each supplementary figure) are available if they are needed. Figure S1: Day 1: 10-20 system EEG: diagnosis of NCSE. Figure S2: Day 1: 10-20 system EEG: burst suppression. Figure S3: Day 2: PDF format generated by BIS VISTA module: burst suppression 4 channels sensor EEG. Figure S4: Day 3: PDF format generated by BIS VISTA module: spikes, spikes waves, and 2-channel sensor. Figure S5: Day 3: 10-20 system EEG: diagnosis of NCSE recurrence. [file 1208401.f1.zip › figure S3_CRICC_2459589.tif]

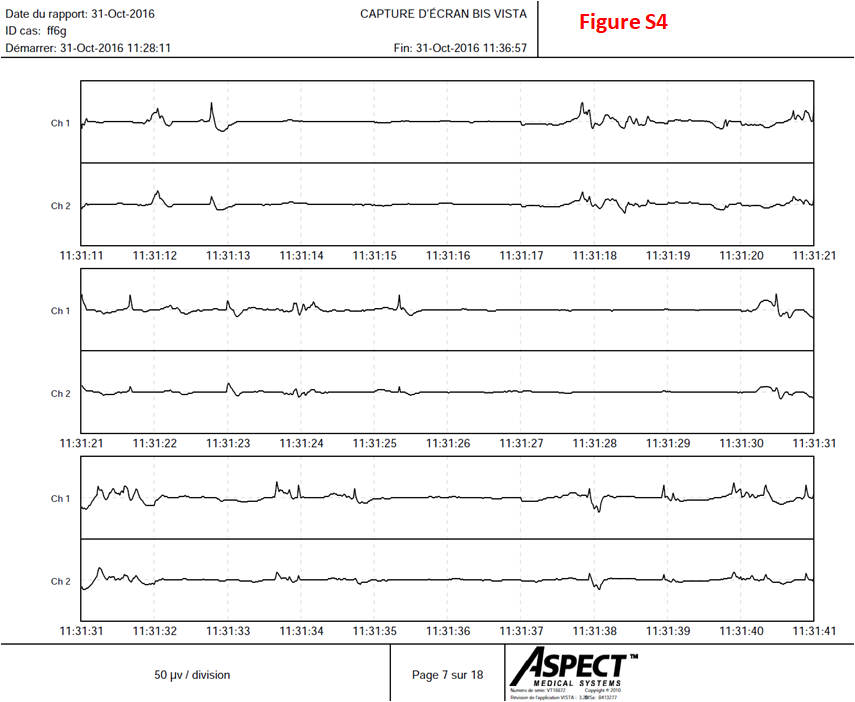

Supplement: Supplementary Materials — I expect the case report to have a high pedagogical value, so there are one principal figure (Figure 1) and one principal video (video 1). There are 5 supplementary figures (figure S1-figure S2-figure S3-figure S4-figure S5): each one is supposed to help the editorial board to confirm the accuracy and the relevance of the case report. Also they will help readers if necessary. (i) Figure S1 is a conventional electroencephalogram recorded at the admission of the patient which confirmed the nonconvulsive status epilepticus. (ii) Figure S2 is as the figure S2, a conventional electroencephalogram recorded few hours later, which confirmed that the nonconvulsive status epilepticus was well treated. (iii) Figure S3 is an electroencephalogram, recorded on day 2, which is displayed by a BISvista monitor connected to a bilateral sensor. (iv) Figure S4, as figure S3, is an electroencephalogram, recorded on day 3, which is displayed by a BISvista monitor connected to unilateral sensor. (v) Figure S5, as figures S1 and S2, is a conventional electroencephalogram recorded on day 3, which confirmed the nonconvulsive status epilepticus recurrence. The complete files (30 minutes EEG recording for each supplementary figure) are available if they are needed. Figure S1: Day 1: 10-20 system EEG: diagnosis of NCSE. Figure S2: Day 1: 10-20 system EEG: burst suppression. Figure S3: Day 2: PDF format generated by BIS VISTA module: burst suppression 4 channels sensor EEG. Figure S4: Day 3: PDF format generated by BIS VISTA module: spikes, spikes waves, and 2-channel sensor. Figure S5: Day 3: 10-20 system EEG: diagnosis of NCSE recurrence. [file 1208401.f1.zip › figure S4_CRICC_2459590.tif]

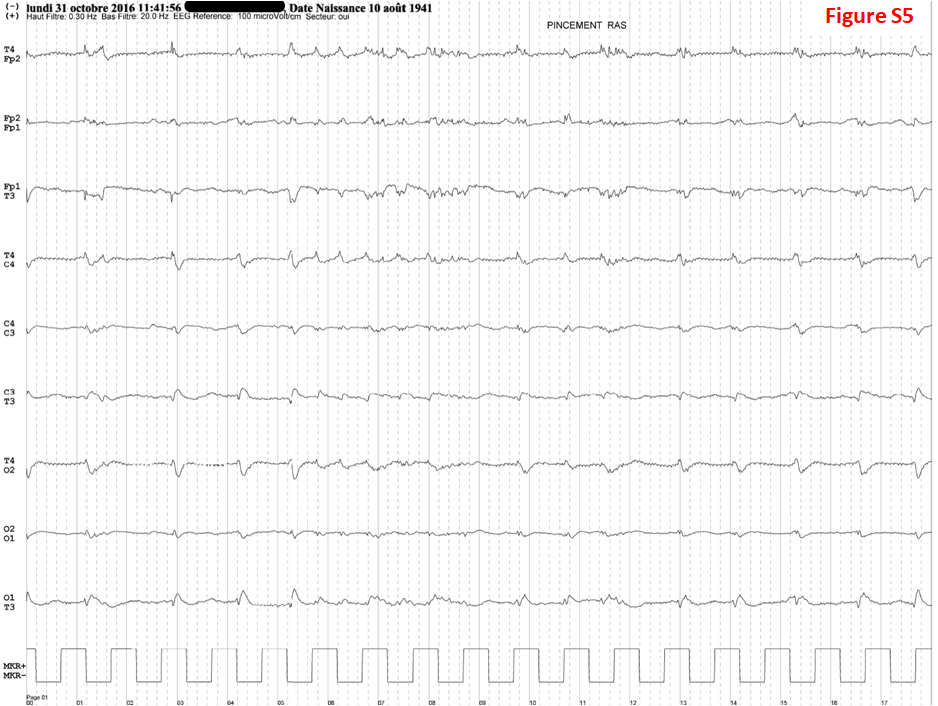

Supplement: Supplementary Materials — I expect the case report to have a high pedagogical value, so there are one principal figure (Figure 1) and one principal video (video 1). There are 5 supplementary figures (figure S1-figure S2-figure S3-figure S4-figure S5): each one is supposed to help the editorial board to confirm the accuracy and the relevance of the case report. Also they will help readers if necessary. (i) Figure S1 is a conventional electroencephalogram recorded at the admission of the patient which confirmed the nonconvulsive status epilepticus. (ii) Figure S2 is as the figure S2, a conventional electroencephalogram recorded few hours later, which confirmed that the nonconvulsive status epilepticus was well treated. (iii) Figure S3 is an electroencephalogram, recorded on day 2, which is displayed by a BISvista monitor connected to a bilateral sensor. (iv) Figure S4, as figure S3, is an electroencephalogram, recorded on day 3, which is displayed by a BISvista monitor connected to unilateral sensor. (v) Figure S5, as figures S1 and S2, is a conventional electroencephalogram recorded on day 3, which confirmed the nonconvulsive status epilepticus recurrence. The complete files (30 minutes EEG recording for each supplementary figure) are available if they are needed. Figure S1: Day 1: 10-20 system EEG: diagnosis of NCSE. Figure S2: Day 1: 10-20 system EEG: burst suppression. Figure S3: Day 2: PDF format generated by BIS VISTA module: burst suppression 4 channels sensor EEG. Figure S4: Day 3: PDF format generated by BIS VISTA module: spikes, spikes waves, and 2-channel sensor. Figure S5: Day 3: 10-20 system EEG: diagnosis of NCSE recurrence. [file 1208401.f1.zip › figure S5_CRICC_2459591.tif]
